# Perceived Comfort and Blinding Efficacy in Randomised Sham-Controlled Transcranial Direct Current Stimulation (tDCS) Trials at 2 mA in Young and Older Healthy Adults

**DOI:** 10.1371/journal.pone.0149703

**Published:** 2016-02-22

**Authors:** Denise Wallace, Nicholas R. Cooper, Silke Paulmann, Paul B. Fitzgerald, Riccardo Russo

**Affiliations:** 1 Department of Psychology and Centre for Brain Science, University of Essex, Colchester, Essex, United Kingdom; 2 Monash Alfred Psychiatry Research Centre, Monash University Central Clinical School and The Alfred, Monash University, Melbourne, Australia; University of Ottawa, CANADA

## Abstract

**Background:**

tDCS studies typically find that: lowest levels of comfort occur at stimulation-onset; young adult participants experience less comfort than older participants; and participants’ blinding seems effective at low current strengths. At 2 mA conflicting results have been reported, questioning the effectiveness of blinding in sham-controlled paradigms using higher current strengths. Investigator blinding is rarely reported.

**Objective:**

Using a protocol with 30 min of 2 mA stimulation we sought to: (a) investigate the level of perceived comfort in young and older adults, ranging in age from 19 to 29 years and 63 to 76 years, respectively; (b) test investigator and participant blinding; (c) assess comfort over a longer stimulation duration; (d) add to the literature on protocols using 2 mA current strength.

**Methods:**

A two-session experiment was conducted where sham and active stimulation were administered to the frontal cortex at the F8/FP1 sites in a within-subjects manner. Levels of perceived comfort were measured, using a visual analogue scale, at the start and end of stimulation in young and older adults. Post-stimulation, participants and investigators judged whether or not active stimulation was used.

**Results:**

Comfort scores were lower at stimulation onset in both age groups. Older adults reported: (i) more comfort than young participants overall; (ii) comparable levels of comfort in sham and active stimulation; (iii) significantly more comfort than the young participants during active stimulation. Stimulation mode was correctly identified above chance in the second of the two sessions; 65% of all participants correctly identified the stimulation mode, resulting in a statistical trend. Similarly, the experimenter correctly identified stimulation mode significantly above chance, with 62% of all investigator judgements correct across 120 judgements.

**Conclusions:**

Using 2 mA current strength over 30 minutes, tDCS stimulation comfort is lower at stimulation onset in young and older adults and, overall, lower for young participants. Investigators and participants may be able to identify active stimulation at above chance levels, although accuracy never exceeded 65% for either participants or the experimenter. Further research into blinding efficacy is recommended.

## Introduction

Transcranial direct current stimulation (tDCS) is a method of neuromodulation that modifies the threshold for neuronal excitation in a polarity-dependent way [1–5]. tDCS has shown potential in both research and clinical applications (for review see [6]). For example, recent research has found that certain cognitive capabilities can be enhanced in healthy [7] and brain-damaged [8] adults. In addition, tDCS has been used to facilitate stroke rehabilitation [9], attenuate addiction [10] and pain [11], treat depression [12] and several other neurological and psychiatric disorders (e.g. [13,14,15]). Parallel to the above developments is the important research into the safety, comfort (i.e. tolerability of electrical stimulation) and blinding robustness of tDCS double-blind sham-controlled trials.

In a recent systematic review of 209 studies (from 172 articles) regarding adverse effects (AEs) associated with tDCS, Brunoni and colleagues (2012) found that tDCS is safe and well tolerated when used for 1 to 2 sessions with healthy participants. The two most commonly reported symptoms were itching and tingling, with no statistically significant differences observed between active and sham conditions, though these symptoms were higher in the active compared to the sham group. Burning sensations and discomfort were reported in a small proportion of participants [1]. A more recent study investigating perceptibility in transcranial electrical stimulation in 693 different sessions found a similar profile of symptoms with pinching and itchiness as the two most commonly reported symptoms [16]. Importantly, Brunoni and colleagues (2012) also found a selective reporting bias where 44% of the 209 studies did not report the presence/absence of AEs, suggesting that continued research in this area is necessary.

Skin irritation and discomfort, which are caused by excitation of peripheral nerves and electrochemical reaction at the electrode-skin interface during electrical stimulation [17], are the most commonly cited issues with tDCS testing [1,3,18,19]. Levels of comfort could also be influenced by additional factors, such as age. Ageing skin tends to be dryer as collagen, elastin and subcutaneous fat levels in the skin decrease [20,21], thereby reducing skin conductance and, arguably, decreasing perceived comfort. However, two sham-controlled studies reporting on perceived comfort during tDCS stimulation [22,23] found that older participants experienced less discomfort during stimulation than young adults. These studies used 1 mA current for 20 minutes and 1.5 mA for 10–20 minutes, respectively. In particular, Kessler and colleagues (2012) found that the proportion of reported sensory symptoms declined with each added year of age. More recently, Fertonani and colleagues (2015) similarly found, using a current strength of 2 mA with 35 cm^2^ electrodes, that older participants reported less discomfort compared to young participants. They used a questionnaire that measured a number of specific symptoms plus an overall discomfort measure to indicate the level of discomfort perceived. These findings are consistent with a recent evoked potentials study investigating age-related sensitivity to electrical stimulation [24]. Regrettably, these tDCS studies provided very little [22] or no [16,23] data regarding participants’ blinding.

Studies investigating blinding robustness at 1 mA current strength, using a standard protocol for sham where stimulation is ramped up and down with a short stimulation duration (i.e. ≤ 30 s), found that participants could not reliably distinguish active from sham stimulation [22,25,26]. These findings correspond to similar outcomes relating to perceived comfort where sensations, such as itching and tingling, reported in active compared to sham conditions were similar [22,23,25,27]. At higher current strengths, such as 2 mA, previous studies show that the disparity between perceived comfort in active and sham condition is greater [23,28,29] and therefore it follows that using a higher current strength is more likely to undermine blinding robustness, particularly in within-subjects designs where both active and sham conditions are administered to each participant. Indeed, two recent within-subjects double-blind sham-controlled studies, using the same current density (2 mA/35 cm^2^ = 0.0571 mA/cm^2^) and delivered over the same duration (20 min), reported conflicting outcomes. O’Connell and colleagues (2012) found that their participants could detect the stimulation received at an above chance level. In contrast, Palm and colleagues (2014) found that participants could not reliably distinguish between active and sham stimulation. There were important methodological differences between the two studies which may have led to the different results. Firstly, O’Connell and colleagues (2012) told participants that both stimulation conditions would be administered, whereas Palm and Colleagues (2014) did not. Secondly, Palm and colleagues (2014) wanted to accentuate the difference between sham and active stimulation, therefore not even a minimal form of active stimulation was given during sham, while O’Connell and colleagues (2012) delivered 30 s active stimulation at the start of the sham condition to mimic the effects of active stimulation. Thirdly, sample size, ramp-up/down times, electrolyte solution and wash-out periods also differed between these studies [29,30]. Further clarification regarding blinding efficacy with this design is therefore needed.

Outcomes on investigators’ blinding seem more consistent. Across different levels of current strengths (1 mA to 2 mA) investigators are better able to discern active compared to sham stimulation than participants, whether as participants themselves [26,31] or as observers [29,30]. The latter results are concerning, particularly in the context of double-blind clinical trials where the researcher may have to interact with the participant following tDCS stimulation. Skin redness appeared to be the only observed reaction that yielded a significant difference between stimulation conditions [29,30].

The present study used a within-subjects double-blind sham-controlled design with two main objectives: a) to compare the perceived comfort levels in healthy young and older adult volunteers at 2 mA; and b) to fully assess blinding robustness in a within-subjects design for participants and the investigator. Thereby, we aim to present a more complete picture of comfort and blinding efficacy in healthy aging adults compared to a young sample. In accordance with our previously published work [28], the above was undertaken using a longer active stimulation phase (30 min compared to ≤ 20 min) and a longer sham stimulation phase (1 min compared to ≤30 s) than typically used in previous comfort studies (e.g., Refs. [22,23,25,29,30]).

## Methods and Materials

The study was approved by the University of Essex Faculty of Science and Engineering Ethics Committee and was conducted in accordance with the Declaration of Helsinki. All participants gave written informed consent.

### Participants

Sixty-four right-handed adults took part in the present study. We recruited participants from two separate age groups: young adults and older adults. Our young adults sample comprised 34 participants (22 female) ranging in age from 19 to 29 years (M 21.9, SD 2.6). Our older adults sample comprised 30 participants (23 female) ranging in age from 63 to 76 years (M 68.6, SD 4). Prior to taking part, all participants completed two screening questionnaires: a brief medical history and a transcranial magnetic stimulation Adult Safety Screen (TASS) with the added question, “Have you ever had an adverse reaction to Transcranial Direct Current Stimulation?” [32]. Applicants were eligible to take part if they confirmed no adverse reaction to transcranial magnetic or direct current stimulation, had no brain injuries, history of neurological disorders, drug addiction or recreational drug use, nor diagnosis of any psychiatric disorder and were not taking medications affecting the central nervous system. We also excluded individuals fitted with electronic medical devices. Older participants also completed the Mini-Mental State Examination (MMSE) [33]. The MMSE is a reliable, 30-item measure of cognitive functioning. Scores ranging from 24 to 30 indicate the absence of cognitive impairment [34]; we excluded participants scoring below 26. Although we report here on the comfort of tDCS stimulation, this study is part of a larger investigation into emotional prosody and in terms of this focus we also administered the DASS-21 (Depression Anxiety Stress Scales) [35]. Research has shown that negative emotional states such as depression can adversely affect the ability to recognise emotions [36]. The DASS-21 was therefore administered to assess whether any participants were severely depressed (i.e. scored higher than 21 on the DASS depression scale). In addition, evidence suggests that anxiety and depression modulate pain perception [37]. From a comfort perspective, the DASS scores would allow us to report any impact of anxiety, depression or stress on perceived comfort during tDCS stimulation. The DASS-21 is a self-report questionnaire measuring depression, anxiety and stress. Each scale comprised 7 items measured on a 4-point rating scale (“Never”– 0 to “Almost Always”– 3) and has a possible score range of 0 to 21. The manual recommends multiplying these scores by 2 to be comparable with the full DASS. Thus, scores reported here range from 0 to 42 per scale with a summed total score of up to 126. We recruited participants from the students’ population on campus and through local newspaper advertisements in Colchester, UK.

### Study design

We used a within-subjects design, where participants received two stimulation conditions, active and sham, over two sessions, at least 7 days apart. We calculated that a sample size of 30 per group would achieve a power of approximately 80% to detect a significant difference between sham and active stimulation assuming a medium effect size (d = 0.5) of the active vs sham manipulation of comfort levels. The order of active and sham stimulation was counterbalanced across participants. The same montage was used in both sessions: right anodal (F8)/left cathodal (FP1). The delivery of the stimulation conditions utilised a double-blind approach. For this purpose, we used the “study mode” of the tDCS stimulator. This mode aims to produce similar sensory experiences to mask the stimulation condition administered. The experimenter used a list of block-randomised codes provided by the principal investigator to execute each stimulation session. The experimenter knew that all participants would receive both conditions, but was blind to the order. The experimenter taped a small cardboard flap over the display window as an extra assurance that none of the information displayed could indicate the mode of stimulation.

### tDCS Stimulation

#### a) Equipment and preparation

We used a DC-Stimulator Plus with a tDCS Equipment Electrode Set (Neuroconn GmbH, Germany). The latter comprised two electrode cables to connect the tDCS stimulator to the electrodes, two 35 cm^2^ conductive rubber electrodes which were inserted into corresponding 35 cm^2^ sponges (composed of 70% regenerated cellulose and 30% cotton). The sponge-electrodes were secured to the head using non-conductive elasticised head straps (up to 5 straps were used to ensure that the sponge-electrodes made good contact with the skin).

The sponge-electrodes were soaked in approximately 20 ml of saline solution. The NaCl concentration used was 100 mM dissolved in distilled water. This concentration was based on the findings of Dundas and colleagues (2007) who recommended using NaCl concentrations ranging from 15 mM to 140 mM NaCl because, within this range, perceived comfort was greater and required relatively low voltage while still producing good current conduction. To apply the saline with a syringe, the sponges were placed on a dry towel, on a clean table. This method ensured that any excess solution was soaked into the towel so that the sponges did not drip when secured to the head. The electrodes were then inserted. The sponges were checked before stimulation began to ensure that they were very moist. If not, more saline solution was carefully re-applied to the sponge/s using the syringe. After each use, the sponges were washed thoroughly and towel-dried. The sponges and electrodes used for this study were new and were regularly inspected for wear/irregularities. The electrodes were positioned according to the International 10–20 system for EEG electrode placement [38]. For this purpose, we used a blank EEG cap with marked-out cross-referenced positions. We also measured the vertex to ensure that our orientation of the cap was correct. The F8 and FP1 positions were close to the edges of the cap, allowing us to mark out the positions with relative ease, using a non-permanent marker. The sponge-electrodes were placed in the centre of the marked position. Prior to measurements of the head being taken, the skin was inspected to ensure that there were no lesions or rashes. The skin was then gently cleaned with an alcohol wipe or damp cloth (patted dry) if preferred.

#### b) Stimulation protocol

In the active stimulation condition, a current strength of 2 mA was applied over 30 min (1800 s) with a ramp-up and ramp-down period of 30 s. We used a longer active stimulation period to accommodate the length of the cognitive tasks undertaken. In addition, we wished to replicate and extend our previous between-subjects findings regarding perceived comfort and blinding using the same testing protocols [28]. In the sham condition, ramp-up duration was 30 s followed by 1 min of 2 mA stimulation then a 30 s ramp-down. The neuroConn DC-Stimulator Plus device calculates the length of active stimulation by dividing the programmed stimulation duration (in seconds) by 30 [39]. Using 1 min of active stimulation is proportionate to the longer active stimulation duration used in this study, compared to other studies which typically use ≤ 30 s active stimulation for a stimulation duration of ≤ 20 min. For the rest of the sham stimulation, the stimulator produced a small current pulse every 550 ms (110 μA over 15 ms). The peak current duration was 3 ms. Average current over time was not more than 2 μA, which has no therapeutic effect [39]. At the start of each tDCS stimulation session the small display window on the stimulator was checked to ensure that the device was working correctly. The stimulator is designed to cut out at 26 V, making a loud beep sound. Participants were not told that they might receive sham stimulation.

### Tasks

#### a) Visual Analogue Scales

Participants completed visual analogue scales (VAS) measuring comfort during stimulation. We adopted the comfort VAS from [28]: participants were asked to indicate how comfortable they felt at that moment by placing an “x” on a horizontal line anchored at 0 cm with “very uncomfortable” and 10 cm with “very comfortable”. Scores ranged from 0 to 10, with a higher score indicating greater comfort. Participants could also choose to describe the nature of discomfort in the open-ended question.

#### b) On/off judgement questionnaire

The on/off judgement questionnaire required participants to answer ‘yes’ or ‘no’ to the question “Do you think that you received active stimulation during your participation in this study?”.

#### c) Cognitive tasks

In addition to comfort monitoring, participants also completed two emotion-based judgement tasks during stimulation. In the first task, participants were asked to look at a range of facial expressions shown on a computer screen and identify the emotion they thought was expressed. In the second task, participants had to listen to a range of pseudo-sentences spoken in different tones of voice and identify the emotion they thought was expressed. Analyses on these tasks are not reported here.

### Procedure

Testing took place in a laboratory at the Department of Psychology, University of Essex, which comprised a single well-lit room containing a desk and two chairs as well as a researcher observation area which faced a sound booth.

**Fig 1:**
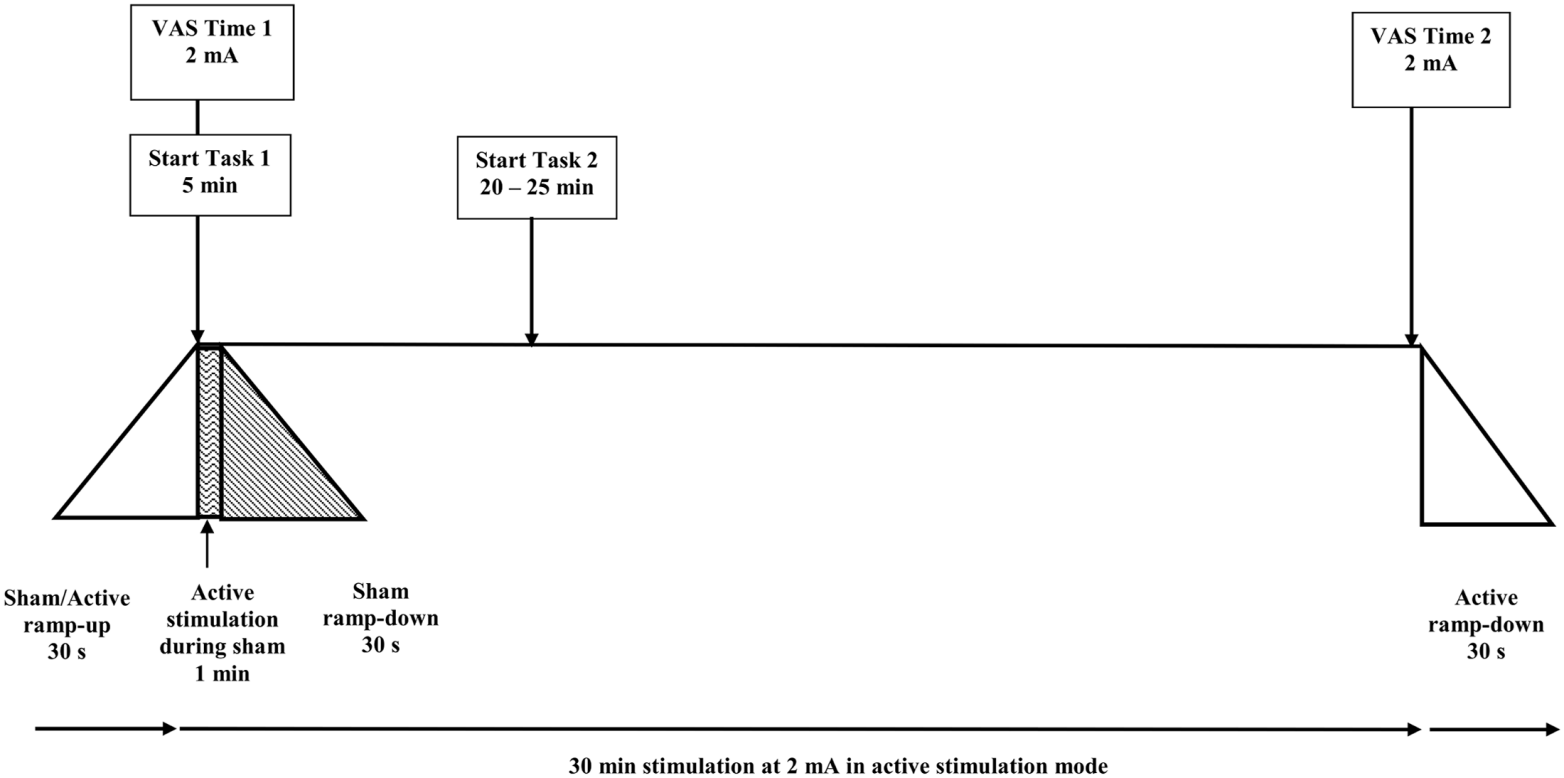
Diagram of the time course for the 30-minute stimulation period at 2 mA. Comfort was measured at the onset of full current strength of stimulation (time 1) and 30 s before the end of stimulation duration (i.e. directly before ramp-down commenced). In the first 5 min of stimulation participants completed Task 1. In the remaining 25 min, participants completed Task 2. The two tasks were completed sequentially, during a continuous period of 30 min tDCS stimulation. Please note: this diagram is not to scale.

Prospective participants were screened either prior to or at session 1. At the outset, participants were required to watch a short video demonstrating the procedure and safety of taking part in tDCS research (https://www.youtube.com/watch?v=hp6bBs16g28) and signed an informed consent form. Participants then completed the DASS-21 questionnaire. All task instructions and procedures were then explained in full. For the duration of the stimulation period participants were seated in front of a computer screen and button box in the sound booth, which had a window and was wired for sound to ensure audio-visual contact throughout testing. The experimenter administered the first VAS (time 1) immediately following the 30 s ramp-up period and the second VAS 30 s (time 2) before ramp-down commenced. The cognitive tasks were completed sequentially, as shown in Fig 1.

At the end of session 2, which followed the same procedure as session 1, each participant completed two on/off judgements; one for each session. The experimenter completed an on/off judgement at the end of each session, out of sight of the participant and before removing the electrodes from the skin. Participants were debriefed at the end of session 2.

### Statistical analysis

We analysed comfort using a mixed analysis of variance (ANOVA). Stimulation condition (active, sham) and time (time 1, time 2) were the within-subjects variables and age group (young vs older adults), the between-subjects variable.

To analyse the on/off judgements two important issues were considered in choosing the most appropriate method of analysis. Firstly, we led participants to expect active stimulation in both sessions, therefore a bias towards selecting ‘yes’ (i.e. ‘on’) was likely to occur. Secondly, participants made two judgements each, but we wanted to know how each participant performed by session and whether participants improved in their accuracy in the second session based on their experience in session 1. We therefore chose to assume that each trial was independent within each session and conducted a binomial test by session on the number of correct trials out of 30 (by age group) and out of 60 (overall sample). Additionally, we applied a McNemar test, comparing the change in the proportion of correct stimulation’s detection between session 1 and session 2 to assess whether there was a significant change in accuracy between sessions.

The experimenter performed 120 judgements in total (60 per age group). We assumed independent trials for these judgements and performed the binomial test on the total trials (n = 120). We also acknowledge that this assumption may be considered too liberal, thus we provide a table with the proportions of correct/incorrect judgements in the active and sham conditions. These data should provide relatively clear indications regarding investigator blinding robustness.

The alpha level was 0.05 and Bonferroni adjustments were applied where appropriate.

## Results

Data from four young female participants were excluded from analysis. One female (age 20) withdrew without giving a reason; one female (age 23) withdrew due to discomfort following session 1. We excluded two further female participants’ data (both age 21), because they had developed a small blister or red mark at the F8 electrode site during testing. In both cases the lesions cleared up completely within 6 days following stimulation.

The final sample analysed comprised 60 participants, 30 in each age group. Twelve young (35%) and 1 (3%) older participants had previously taken part in an unrelated tDCS study at the university, thus non-naïve. We do not know whether they received either sham or active stimulation conditions or both. Older participants’ MMSE scores were ≥ 27; biographical details are presented in Table 1.

**Table 1:** Biographical information of analysed sample.

	young adults (n = 30)	older adults (n = 30)
Mean (SD) age (years)	21.93 (2.7)	68.57 (4.0)
Sex (female; male)	18;12	23;7
Educational level: (up to A-levels^a^; higher education^b^)	0;30	10;20
MMSE (score out of 30)	N/A	≥ 27
Naïve to tDCS (yes; no)	18;12	29;1

^a^ Number of participants who are secondary school leavers.

^b^ Number of participants who continued on to higher education (e.g. university degree, diploma, vocational training)

DASS scores for the two age groups were comparable. Both a one-way ANOVA and Kruskal-Wallis tests was performed with identical results. We conducted a Pearson product-moment correlation on the 3 scales of the DASS-21 and the total score in relation to the time 1 and 2 comfort scores collected during active and sham stimulation. The results indicated that there is no linear association between comfort scores and negative emotional state as measured by the DASS-21 (p’s ≥ 0.1). Table 2 presents the descriptive data and p-values for DASS scores by group.

**Table 2:** DASS-21 mean (standard error) and median scores and p-values, by group.

	Young adults		Older adults		Kruskal-Wallis Test
Scale (score range)	M (SE)	Median (range)	M (SE)	Median (range)	p
Depression (0–42)	4.13 (0.68)	4 (0–12)	3.33 (0.73)	2 (0–18)	> 0.2
Anxiety (0–42)	4.07 (0.92)	3 (0–20)	2.47 (0.57)	2 (0–14)	> 0.2
Stress (0–42)	8.20 (1.34)	6 (0–22)	7.07 (1.05)	6 (0–22)	> 0.2
Total (0–126)	16.40 (2.34)	13 (0–52)	12.87 (2.04)	10 (0–52)	> 0.2

### Comfort

Overall, 28.3% (n = 17/60) of participants scored below 5 on our 0–10 cm VAS comfort scale during active stimulation, whereas the proportion was 13.3% (8/60) during sham. Table 3 presents the descriptive data for age group by stimulation condition and time. We found significant main effects of stimulation condition (*F*(1, 58) = 8.38, *p* <0.01) and time (*F*(1,58) = 107.20, *p* < 0.01) indicating that participants were more comfortable during sham (M 6.78, SE 0.18) than active stimulation (M 6.27, SE 0.22) and that comfort was higher at time 2 (M 7.66, SE 0.19) than time 1 (M 5.39, SE 0.23). The main effect of age was significant (*F*(1,58) = 6.46, *p* < 0.015) with the older group (M 6.98, SE 0.25) reporting higher levels of comfort than young participants (M 6.07, SE 0.25). The stimulation condition by age (*F*(1,58) = 4.72, *p* < 0.035) and stimulation condition by time interactions (*F*(1,58) = 20.82, *p* < 0.01) were significant.

**Table 3:** Descriptives (means and SEs) of age group by stimulation condition and time.

	Active Stimulation		Sham Stimulation		Stimulation Condition	
	Time 1	Time 2	Time 1	Time 2	Active	Sham
**AGE**	M (SE)	M (SE)	M (SE)	M (SE)	M (SE)	M (SE)
19–29 YEARS (n = 30)	4.67 (0.35)	6.57 (0.37)	4.86 (0.38)	8.17 (0.22)	5.62 (0.30)	6.51 (0.23)
63–76 YEARS (n = 30)	6.23 (0.35)	7.60 (0.37)	5.78 (0.38)	8.30 (0.22)	6.91 (0.30)	7.04 (0.28)
TOTAL SAMPLE (n = 60)	5.45 (0.27)	7.09 (0.27)	5.32 (0.27)	8.23 (0.15)	6.27 (0.22)	6.78 (0.18)

Post-hoc follow-up tests were conducted with a Bonferroni-corrected alpha of *p* = 0.008. Simple main effects analyses for the age by stimulation condition interaction revealed that only in the young group were comfort levels significantly lower in the active (M 5.62) than sham (M 6.51) stimulation condition (t(29) = -3.854, p = 0.001); comfort levels were comparable for both stimulation conditions in the older group (M 6.91vs M 7.04) (t(29) = -0.480, p > 0.6). We also compared comfort between young and older groups within each stimulation condition. Independent samples t-tests revealed that, in the active stimulation condition only, comfort levels were significantly lower in the young (M 5.62) than older group (M 6.91) (t(58) -3.005, p = 0.004); scores were comparable during sham (t(58) -1.443, p > 0.1). For the time by stimulation condition interaction effect we compared active with sham stimulation for each time point across groups. Paired-samples t-tests showed that only at time 2 was there a significant difference between stimulation conditions (t(59) -5.143, p < 0.001); comfort scores were lower during active (M 7.09) compared to sham (M 8.23) condition. At time 1 scores were comparable: (t(59) 0.537, p > 0.5). Finally, the time by age group and the stimulation by time by age group interactions were not significant (*F*s < 2.26, *p*s ≥ 0.10).

To assess whether our 13 ‘non-naïve’ participants responded differently to our ‘naïve’ participants, we conducted a mixed ANOVA with stimulation condition (active vs. sham) and time (time 1 vs. time 2) as within-subjects variables and naïve to tDCS (yes vs. no) as between-subjects variable. We found that naïve participants (n = 47) reported levels of comfort comparable to that of non-naïve participants (n = 13) (*F*(1,58) 1.92, *p* > 0.15). Moreover, this factor did not significantly enter in any significant interaction including the other factors (*Fs* < 1.94, *ps* > 0.17).

### On/off judgements

Table 4 presents the on/off judgements for participants compared to actual stimulation conditions for each session, by age group. As expected, the table shows there is a bias among participants towards selecting active stimulation. In both sessions the proportion of participants who believed they were given active stimulation was 85%. For the experimenter, the proportions were 40% and 50% for session 1 and 2, respectively.

**Table 4:** Participant judgements compared to actual stimulation condition, by session by age group.

			Stimulation Condition			Binomial test^a^
Participant judgements			Active	Sham	Totals	p
Session 1	Young	ACTIVE	*15*	14	29	
		SHAM	0	*1*	1	> 0.50
	Older	ACTIVE	*10*	12	22	
		SHAM	5	*3*	8	> 0.50
		Totals	30	30	60	
Session 2	Young	ACTIVE	15	11	26	
		SHAM	0	*4*	4	> 0.10
	Older	ACTIVE	*15*	10	25	
		SHAM	0	*5*	5	> 0.05
		Totals	30	30	60	

Bonferroni correction = 0.05/2 = 0.025

^a^The Binomial test refers to the proportion of correct responses about stimulation conditions per group and per session. Other analyses on data collapsed within sessions or across sessions, where appropriate, are provided in the main text.

Referring to Table 4, session 1, 83% of participants (25/30) who received active stimulation and 13% (4/30) of the sham recipients were correct in their judgements. In session 2, the figures were 100% (30/30) and 30% (9/30), respectively.

The binomial test indicated that in session 1 participants were not able to successfully detect above chance which stimulation condition they received (i.e. 29/60, 48%, *p* > 0.10). However, in session 2 the result showed a trend (i.e. 39/60, 65%, *p* = 0.027, corrected α = 0.025) indicating that participants were able to discern above chance the stimulation condition administered. The McNemar test was performed to assess whether the proportion of successes, in terms of judgements correct, had changed between sessions. However, no significant increase was found (p > 0.50), suggesting that the participants’ experience in session 1 did not affect their session 2 judgements.

The binomial test conducted for age group at each session revealed no statistically significant findings in either the young (53% and 63%, *p*s > 0.05) or the older group (43% and 66%, *p*s > 0.05). A further ancillary analysis showed that non-naïve participants were no more accurate in their session 1 judgements (*p* > .90) than naïve participants (*p* > 0.70). Session 2 judgements were not analysed as all non-naïve participants responded that they had received active stimulation.

Table 5 presents the on/off judgements for the experimenter compared to actual stimulation conditions for each session, by age group. The experimenter correctly identified the stimulation condition delivered in 74 out of a total of 120 trials (62%), which is above chance with the binomial test (*p* < .015). Greater details on the experimenter accuracy in detecting the correct type of stimulation are given in Table 5. Note that accuracy in detecting the correct stimulation mode, both among participants and for the experimenter, never exceeded 65%.

**Table 5:** Experimenter judgements compared to actual stimulation condition, by session by age group.

			Stimulation Condition			Binomial test^a^
Experimenter judgements			Active	Sham	Totals	p
Session 1	Young	ACTIVE	*9*	5	14	
		SHAM	6	*10*	16	> 0.10
	Older	ACTIVE	*6*	4	10	
		SHAM	9	*11*	20	> 0.50
		Totals	30	30	60	
Session 2	Young	ACTIVE	*9*	5	14	
		SHAM	6	*10*	16	> 0.10
	Older	ACTIVE	*10*	6	16	
		SHAM	5	*9*	14	> 0.10
		Totals	30	30	60	

Bonferroni correction = 0.05/2 = 0.025

^a^The Binomial test refers to the proportion of correct responses about stimulation conditions per group and per session. Other analyses on data collapsed within sessions or across sessions, where appropriate, are provided in the main text.

## Discussion

This investigation examined the impact of 2 mA tDCS stimulation over 30 min on comfort and blinding efficacy in young and older healthy adults. The comfort analyses revealed two key findings: First, older adults found both active and sham stimulation to be comparable, with responses in both stimulation conditions towards the ‘comfortable’ end of the comfort scale. By contrast, young adults found active stimulation significantly less comfortable than sham. Second, in the active condition only, young participants were significantly less comfortable than older participants.

In the on/off analyses our two main findings were: First, neither the young nor older participants could accurately detect which stimulation condition they had received in each session. Similarly, the experimenter was unable to accurately judge stimulation condition in each session within each group. Second, in the overall analysis, participants were fairly accurate in their judgements of session 2 with 65% of judgements correct. They were not accurate about session 1. The experimenter was accurate significantly above chance across 120 judgements.

Participants tolerated the stimulation well given that 72% of participants reported overall comfort levels greater than 5 on the 10 cm VAS. By comparison, Dundas and colleagues (2007) found, at 1 mA current strength with a very similar measure (11 point VAS with 0 as “very uncomfortable” and 10 as “very comfortable”), that 58.93% fell within the upper half of the comfort scale (5–10 cm). At 2 mA current strength, using a 5-poing rating scale ranging from “not at all unpleasant” to “highly unpleasant”, Palm and colleagues (2014) found that 50% of participants rated tDCS stimulation as “not at all unpleasant”, 30% as “lightly unpleasant” and 20% as “moderately unpleasant”, their highest reported discomfort rating. Other studies, using a range of current strengths, reported similar results (e.g. [16,22,25,40]).

The age effect for comfort found in the present study is consistent with previous tDCS results [16,22,23] as well as Kemp and colleagues’ (2014) findings. The latter study [24] found evidence showing that reduced sensation in the skin is due to dysfunction of the peripheral/central nervous system and not age-related changes in skin conductance. Thus, there is a real perceptual and/or sensorial difference between young and older adults, which means older participants could tolerate more electrical stimulation at higher strengths possibly putting them at risk of injury as they are less sensitive to the discomfort associated with this type of stimulation. Fertonani and colleagues (2015) argued that other factors may also play a role, such as a lower propensity to complain and greater tolerability of mild discomfort among the elderly population. Given that the results from current perceived comfort tDCS studies are often based on samples of younger adults [25–31], additional research regarding the safety and comfort of elderly participants is advisable. In particular, future studies could include an examination of the propensity to complain given that this aspect has not been explored in tDCS research and may be relevant when testing an elderly sample.

Some participants continued to experience mild discomfort throughout testing during the active condition, given that, at time 2, comfort scores in the active stimulation condition across participants were significantly lower than sham. Our assessment of negative emotional state as administered by the DASS revealed no statistical evidence that responses were correlated with stimulation mode therefore we are confident that participants’ experience of the active tDCS stimulation was not exacerbated by existing levels of depression, anxiety or stress. We also reported two cases of lesions at the F8 electrode site. We had used exactly the same tDCS protocol with an F3/F4 montage in 195 sessions (61% females) in a previous study without any adverse effects [28]. Thus, we speculate that the lesions and continued discomfort during testing were specific to this montage. It is possible that the lesions occurred because of an uneven current distribution across the area of the anode electrode positioned over the F8 site. This current clustering may have been caused by inter-individual differences in the shape of the skull and/or that the elasticated straps were too tightly secured and caused the bottom corner to lift slightly [41]. Fertonani and colleagues (2015) recommend using a net-shaped elastic bandage for uniform adherence to the scalp to ensure consistent contact skin/electrode contact.

Perceptibility and comfort of active stimulation may affect blinding efficacy. Indeed, Fertonani and colleagues (2015) found that anodal stimulation was slightly more perceptible than sham stimulation, independent of current strength. Though not statistically significant, this finding suggests that anodal stimulation may be detectable in a sham-controlled study even when lower current strength is applied. However, previous findings with current strengths ranging from 1 mA to 2 mA do not support this supposition [22,23,28,29]. The present study showed that older participants report more comfort than young participants, and particularly so in the active stimulation condition, indicating that perhaps blinding efficacy is primarily a concern in young participants. Importantly, in our previous study of young adults [28], conducted using the same tDCS protocol as the present study but utilising a between-subjects design, we demonstrated that lower levels of comfort did not compromise blinding. Likewise, in the present study, young participants, despite experiencing lower levels of comfort during active than sham stimulation, could not distinguish, above chance, which stimulation condition was received. These findings suggest that while comfort is an important safety and ethical consideration, it does not, per se, affect blinding efficacy. However, O’Connell and colleagues’ (2012) study, where participants were told that they would be given both stimulation conditions, suggests that blinding effectiveness is linked to participants’ expectations about the tDCS experience, particularly when sham and active stimulation are given. O’Connell and colleagues (2012) recommended the use of de facto masking [42] in tDCS research, where participants are told that they will receive the active treatment condition throughout participation. Participants would therefore be more likely to anticipate some cutaneous sensations during stimulation, as appeared to be the case in the present study. Given that expectations may play a pivotal role in blinding effectiveness, de facto masking may therefore be the most expedient and effective solution, especially at higher current strengths, particularly when the alternative would be longer wash-out periods, pre-screening participants for electrical stimulation thresholds or topical anaesthetics.

In terms of investigator blinding it appears that the experimenter could distinguish active from sham stimulation, albeit being wrong in over 35% of the trials. This outcome suggests that completing investigator judgements prior to removing the electrodes did not improve investigator blinding; a previous study using skin redness as a method of detecting stimulation mode was accurate in 60% of active stimulation cases [30].

It could be argued that our findings are of limited value because we have not used relatively conventional levels of current strength (i.e. ≤ 1.5 mA) and/or stimulation duration (i.e. ≤ 20 min). However, tDCS has a wide range of applications and practitioners use current strengths ranging from < 1 mA to 2 mA over a stimulation duration of a few seconds up to 30 min [6,16,43]. Indeed, research has shown that effects vary depending on the current strength (e.g. [14,44]) as well as the duration of stimulation (e.g. [5,45,46,47]). Irrespective of the potential neurological effects of tDCS, it is not unreasonable to assume that comfort is unrelated to these effects and that higher current strengths and longer stimulation periods are less comfortable than lower current strengths and shorter durations. For this reason, examining comfort at the upper end of the range is likely to be most useful.

With respect to the issue of perceived comfort, there is a range of additional variables that may affect perceived comfort during tDCS stimulation that we did not explore in our study, such as quality of sleep, alcohol and caffeine consumption. Given random allocation of participants to the order of the stimulation conditions, it is unlikely that these variables may have contributed to the outcome we observed. Indeed, our results are consistent with previous research investigating perceived comfort. Future studies may consider a systematic analysis of the impact of the above factors on perceived comfort associated to tDCS stimulation.

Finally, we collected participants’ on/off judgements for both sessions at the end of session 2 to ensure that blinding was not compromised. Had we requested on/off judgements at the end of each session, participants could have been alerted that, despite being instructed that active stimulation would be used in both sessions, the tDCS device was possibly also operated in a sham mode. The impact of our approach, however, may have been that participants were less able to remember their stimulation experience in session 1 compared to session 2, hence the slight disparity in the participants’ judgements between sessions. In addition, our on/off judgement question was phrased as a binary question asking whether or not active stimulation was experienced, not whether active or sham stimulation was experienced. Participants were already primed to expect active stimulation, therefore this choice of wording may have enhanced the bias towards active stimulation in participants’ responses. It is to be noticed, however, that overall accuracy did not differ between participants and the experimenter, despite the latter being aware of the possibility of the delivery of both sham and active stimulation conditions.

## Conclusion

tDCS stimulation is well tolerated, but more so in the older group who reported comparable comfort levels during active and sham stimulation. Older participants may tolerate electrical stimulation better, because of sub-optimal processing of perceptual and/or sensorial information, which means they may be at a slightly increased risk of harm whilst undergoing tDCS stimulation. While neither the young nor older sample could distinguish active from sham stimulation, 65% of participants in session 2 correctly judged stimulation mode suggesting that blinding may have been compromised. Moreover, the experimenter correctly identified the stimulation condition at significantly above chance levels even though judgements were completed before removing the electrodes, thus skin redness was not a factor. Hence we recommend: (a) additional research on perceived comfort for volunteers over 60 years of age; (b) participants’ expectations should be a key consideration when designing sham-controlled double-blind tDCS studies to ensure successful participants’ blinding; (c) experimenter blinding success requires on-going investigation.

## Supporting Information

S1 Dataset Comfort and blinding dataset. The comfort and blinding dataset contains all the data that we analysed and presented in this report.
(XLSX)

## References

[1] Brunoni AR, Nitsche MA, Bolognini N, Bikson M, Wagner T, Merabet L, et al Clinical research with transcranial direct current stimulation (tDCS): Challenges and future directions. Brain Stimul. 2012;5(3): 175–95. 10.1016/j.brs.2011.03.002 22037126 PMC3270156

[2] Zaghi S, Acar M, Hultgren B, Boggio PS, Fregni F. Noninvasive brain stimulation with low-intensity electrical currents: putative mechanisms of action for direct and alternating current stimulation. The Neuroscientist. 2009;16(3): 285–307. 10.1177/1073858409336227 20040569

[3] Nitsche MA, Cohen LG, Wassermann EM, Priori A, Lang N, Antal A, et al Transcranial direct current stimulation: State of the art 2008. Brain Stimul. 2008;1(3): 206–23. 10.1016/j.brs.2008.06.004 20633386

[4] Bindman LJ, Lippold OCJ, Redfearn JWT. Long-lasting changes in the level of the electrical activity of the cerebral cortex produced by polarizing currents. Nature. 1962;196(4854): 584–5. 13968314 10.1038/196584a0

[5] Nitsche MA, Paulus W. Excitability changes induced in the human motor cortex by weak transcranial direct current stimulation. J Physiol. 2000;527(3): 633–9. 10990547 10.1111/j.1469-7793.2000.t01-1-00633.x PMC2270099

[6] Nitsche MA, Paulus W. Transcranial direct current stimulation—update 2011. Restor Neurol Neurosc. 2011;29(6): 463–92. 10.3233/RNN-2011-0618 22085959

[7] Richmond L, Wolk D, Chein J, Olson IR. Transcranial direct current stimulation enhances verbal working memory training performance over time and near-transfer outcomes. J Cogn Neurosci. 2014 7: 1–12. 24742190 10.1162/jocn_a_00657

[8] Ulam F, Shelton C, Richards L, Davis L, Hunter B, Fregni F, et al Cumulative effects of transcranial direct current stimulation on EEG oscillations and attention/working memory during subacute neurorehabilitation of traumatic brain injury. Clin Neurophysiol. 2015;126(3): 486–9. 10.1016/j.clinph.2014.05.015 24947595

[9] Liew S-L, Santarnecchi E, Buch E, Cohen L. Non-invasive brain stimulation in neurorehabilitation: Local and distant effects for motor recovery. Front Hum. Neurosci. 2014 6;8: 1–8. 25018714 10.3389/fnhum.2014.00378 PMC4072967

[10] Meng Z, Liu C, Yu C, Ma Y. Transcranial direct current stimulation of the frontal-parietal-temporal area attenuates smoking behavior. J Psychiatr Res. 2014;54: 19–25. 10.1016/j.jpsychires.2014.03.007 24731752

[11] Ngernyam N, Jensen MP, Arayawichanon P, Auvichayapat N, Tiamkao S, Janjarasjitt S, et al The effects of transcranial direct current stimulation in patients with neuropathic pain from spinal cord injury. Clin Neurophysiol. 2015;126(2): 382–90. 10.1016/j.clinph.2014.05.034 25027640

[12] Shiozawa P, Fregni F, Benseñor IM, Lotufo PA, Berlim MT, Daskalakis JZ, et al Transcranial direct current stimulation for major depression: an updated systematic review and meta-analysis. Int J Neuropsychopharmacol. 2014;17: 1443–1452. 10.1017/S1461145714000418 24713139

[13] San-juan D, Morales-Quezada L, Orozco Garduño AJ, Alonso-Vanegas M, González-Aragón MF, Espinoza López DA, Vázquez Gregorio R, Anschel DJ, Fregni F. Transcranial direct current stimulation in epilepsy. Brain Stimul. 2015;8(3): 455–464. 10.1016/j.brs.2015.01.001 25697590

[14] Hoy KE, Arnold SL, Emonson MRL, Daskalakis ZJ, Fitzgerald PB. An investigation into the effects of tDCS dose on cognitive performance over time in patients with schizophrenia. Schizophr Res. 2014;155(1–3): 96–100. 10.1016/j.schres.2014.03.006 24703529

[15] Monti A, Ferrucci R, Fumagalli M, Mameli F, Cogiamanian F, Ardolino G, Priori A. Transcranial direct current stimulation (tDCS) and language. J Neurol Neurosurg Psychiatry. 2013;84(8): 832–842. 10.1136/jnnp-2012-302825 23138766 PMC3717599

[16] Fertonani A, Ferrari C, Miniussi C. What do you feel if I apply transcranial electric stimulation? Safety, sensations and secondary induced effects. Clin Neurophysiol. 2015 4; 10.1016/j.clinph.2015.03.015 25922128

[17] Minhas P, Datta A, Bikson M. Cutaneous perception during tDCS: Role of electrode shape and sponge salinity. Clin Neurophysiol. 2011;122(4): 637–8. 10.1016/j.clinph.2010.09.023 21075048 PMC3053077

[18] Bikson M, Datta A, Elwassif M. Establishing safety limits for transcranial direct current stimulation. Clin Neurophysiol. 2009;120(6): 1033–4. 10.1016/j.clinph.2009.03.018 19394269 PMC2754807

[19] Davis NJ, Gold E, Pascual-Leone A, Bracewell RM. Challenges of proper placebo control for non-invasive brain stimulation in clinical and experimental applications. Eur J Neurosci. 2013;38(7): 2973–7. 10.1111/ejn.12307 23869660

[20] Heft MW, Robinson ME. Age differences in suprathreshold sensory function. Age. 2014;36(1): 1–8. 10.1007/s11357-013-9536-9 23625154 PMC3889875

[21] Smith L. Histopathologic characteristics and ultrastructure of aging skin. Cutis. 1989;43(5): 414–24. 2721240

[22] Gandiga PC, Hummel FC, Cohen LG. Transcranial DC stimulation (tDCS): a tool for double-blind sham-controlled clinical studies in brain stimulation. Clin Neurophysiol. 2006;117(4): 845–850. 16427357 10.1016/j.clinph.2005.12.003

[23] Kessler SK, Turkeltaub PE, Benson JG, Hamilton RH. Differences in the experience of active and sham transcranial direct current stimulation. Brain Stimul. 2012;5: 155–162. 10.1016/j.brs.2011.02.007 22037128 PMC3270148

[24] Kemp J, Després O, Pebayle T, Dufour A. Age-related decrease in sensitivity to electrical stimulation is unrelated to skin conductance: An evoked potentials study. Clin Neurophysiol. 2014;125(3): 602–7. 10.1016/j.clinph.2013.08.020 24070673

[25] Poreisz C, Boros K, Antal A, Paulus W. Safety aspects of transcranial direct current stimulation concerning healthy subjects and patients. Brain Res Bull. 2007;72(4–6): 208–214. 17452283 10.1016/j.brainresbull.2007.01.004

[26] Ambrus GG, Al-Moyed H, Chaieb L, Sarp L, Antal A, Paulus W. The fade-in—short stimulation—fade out approach to sham tDCS—reliable at 1 mA for naive and experienced subjects, but not investigators. Brain Stimul. 2012;5(4): 499–504. 10.1016/j.brs.2011.12.001 22405745

[27] Dundas JE, Thickbroom GW, Mastaglia FL. Perception of comfort during transcranial DC stimulation: effect of NaCl solution concentration applied to sponge electrodes. Clin Neurophysiol. 2007;118(5): 1166–1170. 17329167 10.1016/j.clinph.2007.01.010

[28] Russo R, Wallace D, Fitzgerald PB, Cooper NR. Perception of comfort during active and sham transcranial direct current stimulation: a double blind study. Brain Stimul. 2013;6(6): 946–51. 10.1016/j.brs.2013.05.009 23835166

[29] Palm U, Reisinger E, Keeser D, Kuo M-F, Pogarell O, Leicht G, et al Evaluation of sham transcranial direct current stimulation for randomized, placebo-controlled clinical trials. Brain Stimul. 2014;6(4): 690–5. 10.1016/j.brs.2013.01.005 23415938

[30] O’Connell NE, Cossar J, Marston L, Wand BM, Bunce D, Moseley GL, et al Rethinking clinical trials of transcranial direct current stimulation: participant and assessor blinding is inadequate at intensities of 2 mA. PLoS One. 2012;7: e47514 10.1371/journal.pone.0047514 23082174 PMC3474749

[31] Ambrus GG, Paulus W, Antal A. Cutaneous perception thresholds of electrical stimulation methods: Comparison of tDCS and tRNS. Clin Neurophysiol. 2010;121(11): 1908–14. 10.1016/j.clinph.2010.04.020 20471313

[32] Keel JC, Smith MJ, Wassermann EM. A safety screening questionnaire for transcranial magnetic stimulation. Clin Neurophysiol. 2001;112(4): 720 11332408 10.1016/s1388-2457(00)00518-6

[33] Folstein MF, Folstein SE, McHugh PR. "Mini-mental state": a practical method for grading the cognitive state of patients for the clinician. J Psychiat Res. 1975;12(3): 189–98. 1202204 10.1016/0022-3956(75)90026-6

[34] Tombaugh TN, McIntyre NJ. The mini-mental state examination: a comprehensive review. J Am Geriatr Soc. 1992;40(9): 922–935. 1512391 10.1111/j.1532-5415.1992.tb01992.x

[35] Lovibond SH, Lovibond PF. Manual for the Depression Anxiety Stress Scales. 2 ed Sidney: Psychology Foundation; 1995.

[36] Tracey I, Mantyh PW. The cerebral signature for pain perception and its Modulation. Neuron. 2007;55(3):377–91. 17678852 10.1016/j.neuron.2007.07.012

[37] Péron J, El Tamer S, Grandjean D, Leray E, Travers D, Drapier D, et al Major depressive disorder skews the recognition of emotional prosody. Prog Neuropsychopharmacol Biol Psychiatry. 2011;35(4):987–96. 10.1016/j.pnpbp.2011.01.019 21296120

[38] Jasper HH. The ten-twenty electrode system of the International Federation. Electroencephalogr Clin Neurophysiol. 1958(10): 371–5. 10590970

[39] neuroConn GmbH. neuroConn Programmable Direct Current Stimulator (PLUS version) user’s manual. V2.2.0. Created on 12/12/2012.

[40] Turi Z, Ambrus GzG, Ho K-A, Sengupta T, Paulus W, Antal A. When size matters: large electrodes induce greater stimulation-related cutaneous discomfort than smaller electrodes at equivalent current density. Brain Stimul. 2014;7(3): 460–467. 10.1016/j.brs.2014.01.059 24582373

[41] Horvath JC, Carter O, Forte JD. Transcranial direct current stimulation: five important issues we aren't discussing (but probably should be). Front Syst Neurosci. 2014 1;8: 1–8. 24478640 10.3389/fnsys.2014.00002 PMC3901383

[42] Berger VW. De facto masking and other measures to prevent contamination. J Clin Epidemiol. 2012;65(11): 1236 10.1016/j.jclinepi.2012.04.016 23017642

[43] Jo JM, Kim YH, Ko MH, Ohn SH, Joen B, Lee KH. Enhancing the working memory of stroke patients using tDCS. Am J Phys Med Rehabil. 2009;88(5): 404–9. 10.1097/PHM.0b013e3181a0e4cb 19620953

[44] Iyer MB, Mattu U, Grafman J, Lomarev M, Sato S, Wassermann EM. Safety and cognitive effect of frontal DC brain polarization in healthy individuals. Neurology. 2005;64(5): 872–5. 15753425 10.1212/01.WNL.0000152986.07469.E9

[45] Monte-Silva K, Kuo M-F, Hessenthaler S, Fresnoza S, Liebetanz D, Paulus W, et al Induction of late LTP-like plasticity in the human motor cortex by repeated non-invasive brain stimulation. Brain Stimul. 2013;6(3): 424–32. 10.1016/j.brs.2012.04.011 22695026

[46] Batsikadze G, Moliadze V, Paulus W, Kuo MF, Nitsche MA. Partially non-linear stimulation intensity-dependent effects of direct current stimulation on motor cortex excitability in humans. J Physiol. 2013;591(7): 1987–2000. 23339180 10.1113/jphysiol.2012.249730 PMC3624864

[47] Ohn SH, Park CI, Yoo WK, Ko MH, Choi KP, Kim GM, et al Time-dependent effect of transcranial direct current stimulation on the enhancement of working memory. NeuroReport. 2008;19(1): 43–7. 10.1097/WNR.0b013e3282f2adfd 18281890

